# Characterization and LDL‐C management in a cohort of high and very high cardiovascular risk patients: The PORTRAIT‐DYS study

**DOI:** 10.1002/clc.24183

**Published:** 2023-11-06

**Authors:** Cristina Gavina, Daniel Seabra‐Carvalho, Carlos Aguiar, Anastassia Anastassopoulou, Carla Teixeira, Jorge A. Ruivo, Élia Almeida, Leonor Luz‐Duarte, Ana Corte‐Real, Mariana Canelas‐Pais, Tiago Taveira‐Gomes

**Affiliations:** ^1^ Hospital Pedro Hispano ‐ Unidade Local de Saúde de Matosinhos Portugal; ^2^ Department of Medicine, Faculty of Medicine Porto University Porto Portugal; ^3^ UnIC, Faculty of Medicine Porto University Porto Portugal; ^4^ Advanced Heart Failure Unit, Department of Cardiology Hospital Santa Cruz, CHLO Lisbon Portugal; ^5^ Value and Access Daiichi Sankyo Europe GmbH Germany; ^6^ Medical Affairs Daiichi Sankyo Portugal Porto Salvo Portugal; ^7^ Department of Medicine, Lisbon Medical School Lisbon University Portugal; ^8^ Centro Cardiovascular Universidade Lisboa Lisbon University Portugal; ^9^ Value and Access Daiichi Sankyo Portugal Porto Salvo Portugal; ^10^ UCSP Cinfães ACeS Baixo Portugal; ^11^ MTG Research and Development Lab Porto Portugal; ^12^ UCSP Barroselas Unidade Local de Saúde do Alto Minho Portugal; ^13^ Department of Community Medicine, Health Information and Decision, Faculty of Medicine University of Porto Porto Portugal; ^14^ Faculty of Health Sciences Fernando Pessoa University Portugal

**Keywords:** atherosclerosis, cardiovascular risk, cholesterol, computerized, hypolipidemic agents, LDL, medical records systems

## Abstract

**Aim:**

This study aims to characterize sociodemographic and clinical characteristics, use of lipid‐lowering therapies (LLTs), and low‐density lipoprotein cholesterol (LDL‐C) control in a population with increased cardiovascular (CV) risk.

**Methods:**

A cross‐sectional observational study that uses electronic health records of patients from one hospital and across 14 primary care health centers in the North of Portugal, spanning from 2000 to 2020 (index date). Patients presented at least (i) 1 year of clinical data before inclusion, (ii) one primary care appointment 3 years before the index date, and (iii) sufficient data for CV risk classification. Patients were divided into three cohorts: high CV risk; atherosclerotic cardiovascular disease (ASCVD) risk equivalents without established ASCVD; evidence of ASCVD. CV risk and LDL‐C control were defined by the 2019 and 2016 European Society of Cardiology (ESC)/European Atherosclerosis Society (EAS) dyslipidemia guidelines.

**Results:**

A total of 51 609 patients were included, with 23 457 patients classified as high CV risk, 19 864 with ASCVD equivalents, and 8288 with evidence of ASCVD. LDL‐C control with 2016 ESC/EAS guidelines was 32%, 10%, and 18% for each group, respectively. Considering the ESC/EAS 2019 guidelines control level was even lower: 7%, 3%, and 7% for the same cohorts, respectively. Patients without any LLT prescribed ranged from 37% in the high CV risk group to 15% in patients with evidence of ASCVD.

**Conclusion:**

We found that LDL‐C control was very low in patients at higher risk of CV events. An alarming gap between guidelines on dyslipidemia management and clinical implementation persists, even in those at very high risk or with established ASCVD.

## Graphical abstract

We analyzed electronic health records of 51 609 patients from one hospital and 14 primary care centers in Portugal, assessing sociodemographic and clinical characteristics, lipid‐lowering therapies usage, and low‐density lipoprotein cholesterol (LDL‐C) control. Our findings show suboptimal LDL‐C control in high‐risk patients, emphasizing the need for improved strategies.

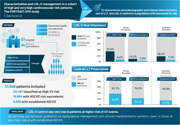

## 1 INTRODUCTION

Atherosclerotic cardiovascular disease (ASCVD) is the leading cause of death and disability worldwide, responsible for over 4 million deaths annually in Europe.^1^, ^2^, ^3^ In Portugal, the main causes of death are ischemic heart disease (126 deaths per 100 000 individuals) and cerebrovascular disease (121 deaths per 100 000 individuals),^4^ represents a significant burden of disability‐adjusted life years (DALYs), with ischemic heart disease accounting 1825 DALYs per 100 000 people and 1738 DALYs per 100 000 people for cerebrovascular disease.^5^ Costa et al. highlight a substantial economic burden with an estimated total cost of €1.9 billion in 2016, corresponding to 1% of gross domestic product and 11% of total health expenditure.^6^

Considering the burden of ASCVD, it is essential to control its risk factors. Dyslipidemia is a well‐established ASCVD risk factor, and high levels of low‐density lipoprotein cholesterol (LDL‐C) are closely related to atheroma plaque progression.^1^ The linear relationship between elevated LDL‐C and ASCVD has been well documented, and lowering LDL‐C, even when its levels are already low, allows a safe reduction of ASCVD risk without a threshold for clinical benefit.^1^, ^7^, ^8^, ^9^, ^10^, ^11^, ^12^ European Society of Cardiology (ESC)/European Atherosclerosis Society (EAS) guidelines for the management of dyslipidemias recommend risk‐based LDL‐C goals, trying to mitigate the absolute risk by achieving the lowest LDL‐C levels in the highest‐risk patients.^3^

The 2019 ESC/EAS guidelines set the LDL‐C goal of <1.4 mmol/L (<55 mg/dL) for individuals at very‐high cardiovascular (CV) risk without familial hypercholesterolemia and an LDL‐C goal of <1.8 mmol/L (<70 mg/dL) for patients at high CV risk. For both, a simultaneous LDL‐C reduction of ≥50% from baseline is also recommended. For patients with established ASCVD who experience a second vascular event within 2 years on maximally tolerated statin therapy, an LDL‐C goal of <1.0 mmol/L (<40 mg/dL) should be considered. The achievement of these LDL‐C goals outside of clinical trials is challenging, and therefore, it is essential to understand how patients can be helped to obtain their LDL‐C goals in clinical practice.^3^

Despite these clinical recommendations across Europe, a gap between clinical guidelines and clinical practice for the management of dyslipidemia persists.^13^, ^14^ The aim of this study was to describe clinical characteristics, lipid‐lowering therapy (LLT) usage, and LDL‐C control in patients at high risk of CV events or ASCVD risk equivalent in primary prevention or patients with established ASCVD in secondary prevention.

## 2 METHODS

### 2.1 Study design

An observational, cross‐sectional methodology was used in this study, analyzing electronic health records (EHR) of patients followed between January 1, 2000 and December 31, 2020 (index date) at the Unidade Local de Saúde de Matosinhos (ULSM). ULSM is a large healthcare institution incorporating one hospital and 14 primary care health units, all supported by the same secondary care health unit at Hospital Pedro Hispano. Patients presented at least (i) 1 year of clinical data before inclusion, (ii) one primary care appointment 3 years before the index date, and (iii) sufficient data for CV risk classification. In total, 136 899 patients were included, which corresponds to about 90% of the Matosinhos region's adult population, based on the 2021 Portuguese Census. Matosinhos is the country's eighth most populous municipality and the fourth in the North region. A 20‐year period of data analyses was applied. The study was approved by the Data Protection Officer and the Ethical Committee of the ULSM (translated from “Comissão de Ética para a Saúde da Unidade Local de Saúde de Matosinhos”).

### 2.2 Patient characterization

All pertinent conditions were identified using the corresponding codes from the International Classification of Disease, 9th Revision (ICD‐9) and 10th Revision (ICD‐10), and the International Classification of Primary Care, version 2 (ICPC‐2). Moreover, current diabetes and CV medications were recorded using the Anatomical Therapeutic Chemical (ATC) Classification System. All patient risk assessment criteria and conditions were attributed using the most granular clinical measurements and laboratory results records available at the ULSM. Familial hypercholesterolemia was classified as possible or definite according to Simon and Broome's criteria since we had no DNA‐based evidence. These criteria account for cholesterol levels, clinical characteristics, molecular diagnosis, and family history, including the risk of fatal coronary heart disease in familial hypercholesterolemia.^15^, ^16^ We used data from primary care family records to establish familial relationships and compute the family history of pertinent diseases. The ankle–brachial index, as well as carotid or coronary imaging data, were not available and, therefore, not incorporated into this study. Observational Medical Outcomes Partnership Common Data Model version 5.3^17^ was used to harmonize source data from EHR. The Supporting Information Materials (S1–S5) contain definitions for all the variables used in this study.

### 2.3 Cohorts

Three cohort groups were defined based on CV risk and the presence of ASCVD equivalents or established ASCVD. The first group included patients with high CV risk. The second group included patients with ASCVD risk equivalents, corresponding to those that meet at least one very‐high CV risk criteria but have no evidence of established ASCVD. This includes the presence of at least one of the following: (i) three major CV risk factors; (ii) estimated glomerular filtration rate <30 mL/min/1.73 m^2^; (iii) Type 1 diabetes duration >20 years; (iv) SCORE value ≥ 10%; (v) familial hypercholesterolemia with a major CV risk factor. The third group included patients with evidence of ASCVD, defined as those who had a diagnosis of unstable angina, myocardial infarction, ischemic stroke, or peripheral artery disease (PAD), as identified using International Classification of Diseases (ICD) codes 9 and 10.

The second group was created to distinguish the very high‐risk patients without evidence of ASCVD, still in primary prevention and more likely followed by their primary care physician, from those that have established ASCVD, already in secondary prevention and frequently followed in‐hospital.

CV risk categories were defined by combining the SCORE and the risk classifications based on morbidity to generate the composite of 2019 and 2016 ESC/EAS guidelines.^3^, ^18^

Among the patients with evidence of ASCVD, a subanalysis was conducted to differentiate between those exclusively managed by primary care physicians and those with regular follow‐up visits by cardiology.

### 2.4 LLT, LDL‐C levels, and LDL‐C goal attainment

We determined a patient's LDL‐C level based on the most recent laboratory test before the index date. In cases where LDL‐C values were missing, the Friedewald formula was used to compute them.^19^ For each CVD risk category analyzed, we assessed the percentage of patients receiving any form of LLT, specifically those prescribed statins, ezetimibe, statin and ezetimibe combination, fibrates, and other treatments. We also evaluated the intensity of the statin therapy, categorized as high, moderate, or low.

We evaluated the LDL‐C goal attainment according to the ESC/EAS 2016 (<100 mg/dL for high CV risk and <70 mg/dL for very‐high CV risk) and the ESC/EAS 2019 (<70 mg/dL for high CV risk and <55 mg/dL for very‐high CV risk) guidelines among the three cohorts studied: High CV risk, ASCVD risk equivalents and evidence of ASCVD.^3^, ^18^ The latter two cohorts meet the very‐high CV risk criteria and thus were subject to the corresponding LDL‐C goal. The proportion of patients attaining the ESC/EAS 2016 and 2019 LDL‐C goals was also computed for each LLT category.

### 2.5 Exposure to LLT

We calculated LLT exposure from prescription records documented by ULSM physicians across primary and secondary care. LLT categories were derived for each individual drug using the ATC Classification System. The American College of Cardiology and the American Heart Association^20^ classification was used to determine the intensity group, taking into account both the drug and the dosage. If a patient had been prescribed an LLT drug within 365 days from the index date, we assumed that the patient was under treatment for that particular LLT category.

### 2.6 Statistical analysis

For continuous variables, we reported the median and the interquartile range (IQR), while for categorical variables, we presented the absolute and relative frequencies. Analyses were descriptive without any comparison between the groups. The final dataset was obtained from the source data using Apache Spark Framework version 2.4.5, the statistical analysis was performed with R version 4.0.3, and Vega‐lite was used for the creation of figures.^21^

## 3 RESULTS

**Figure 1:**
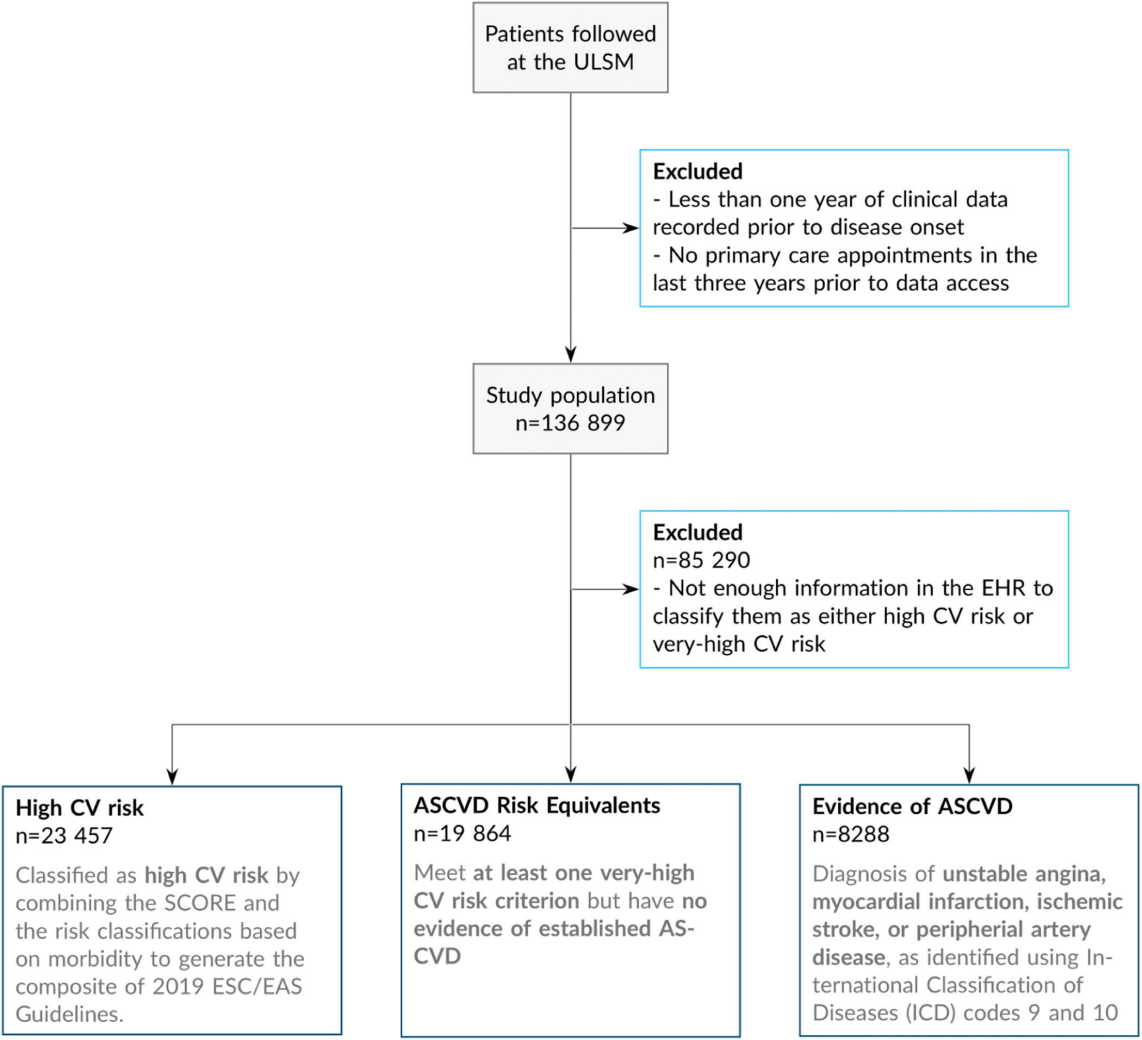
Cohort attrition diagram. ASCVD, atherosclerotic cardiovascular disease; CV, cardiovascular; EAS, European Atherosclerosis Society; EHR, electronic health records; ESC, European Society of Cardiology; ICD‐9, International Classification of Diseases, version 9; ICD‐10, International Classification of Diseases, version 10; SCORE, systematic coronary risk evaluation; ULSM, Unidade Local de Saúde de Matosinhos.

From the 136 899 eligible patients, a total of 51 609 were included, with 23 457 patients classified as high CV risk, 19 864 with ASCVD equivalents, and 8288 with evidence of ASCVD. Figure 1 shows the cohort attrition diagram.

### 3.1 Patient characteristics

Female patients predominated among those with a high CV risk (55%), representing a slightly lower proportion in the ASCVD equivalents (50%) and evidence of ASCVD (48%) cohorts.

The median age was higher among those with evidence of ASCVD (74 years, IQR = 20 years) compared to those with ASCVD equivalents (70 years, IQR = 18 years) and high CV risk (69 years, IQR = 18 years). The proportion of individuals with hypertension and Type 2 DM was higher in ASCVD equivalents (88% and 82%, respectively) and established ASCVD (81% and 64%) cohorts than among those with high‐CV risk (65% and 44%). Table 1 provides further information on the sociodemographic and clinical characteristics of each cohort.

**Table 1:** Sociodemographic and clinical characteristics stratified by CV risk and the presence of ASCVD equivalents at the index date.

	Primary prevention		Secondary prevention
	High CV risk	ASCVD risk equivalence	Evidence of ASCVD
Eligible population, *n* (%)	23 457 (17.1%)	19 864 (14.5%)	8288 (6.1%)
Female, *n* (%)	12 845 (54.8%)	9907 (49.9%)	3978 (48.0%)
Age, years, P50 (IQR)	69 (18)	70 (18)	74 (20)
Waist, cm, P50 (IQR)	98 (14)	101 (15)	100 (16)
Body mass index, kg/m^2^, P50 (IQR)	27 (6)	28 (7)	27 (6)
LDL‐C control, ESC 16, *n* (%)	7591 (32.4%)	2026 (10.2%)	1521 (18.4%)
LDL‐C control, ESC 19, *n* (%)	1544 (6.6%)	672 (3.4%)	590 (7.1%)
LDL‐C, mg/dL, P50 (IQR)	114 (50)	115 (50)	97 (48)
LDL‐C, mmol/dL, P50 (IQR)	2.95 (1.29)	2.97 (1.29)	2.51 (1.24)
Smoking status	*N* (%)	*N* (%)	*N* (%)
Never	18 587 (79.2%)	14 370 (72.3%)	6210 (74.9%)
Current	2293 (9.8%)	3778 (19.0%)	954 (11.5%)
Former	1559 (6.6%)	1190 (6.0%)	696 (8.4%)
General comorbidities	*N* (%)	*N* (%)	*N* (%)
Obesity	6080 (25.9%)	6584 (33.1%)	2096 (25.3%)
Hypercholesterolemia	10 468 (44.6%)	10 561 (53.2%)	2321 (28.0%)
Type 2 diabetes	10 278 (43.8%)	16 367 (82.4%)	5295 (63.9%)
Structural heart disease	2437 (10.4%)	5141 (25.9%)	5257 (63.4%)
Microvascular disease	911 (3.9%)	2000 (10.1%)	1265 (15.3%)
Familial hypercholesterolemia	27 (0.1%)	783 (3.9%)	209 (2.5%)
Cardiovascular comorbidities	*N* (%)	*N* (%)	*N* (%)
Hypertension	15 218 (64.9%)	17 485 (88.0%)	6717 (81.0%)
Atrial fibrillation	1040 (4.4%)	1979 (10.0%)	1805 (21.8%)
Chronic kidney disease	2570 (11.0%)	4860 (24.5%)	2352 (28.4%)
ASCVD	0 (0.0%)	0 (0.0%)	8288 (100.0%)
Unstable angina	0 (0.0%)	0 (0.0%)	422 (5.1%)
Myocardial infarction	0 (0.0%)	0 (0.0%)	2548 (30.7%)
Stroke	80 (0.3%)	142 (0.7%)	5466 (66.0%)
Peripheral artery disease	0 (0.0%)	0 (0.0%)	1096 (13.2%)
Lipid‐lowering therapies	*N* (%)	*N* (%)	*N* (%)
No LLT	8662 (36.9%)	4581 (23.1%)	1261 (15.2%)
Statin without ezetimibe usage	13 377 (57.0%)	13 378 (67.3%)	6208 (74.9%)
High intensity without ezetimibe	1027 (4.4%)	1706 (8.6%)	1129 (13.6%)
Moderate intensity without ezetimibe	11 816 (50.4%)	11 264 (56.7%)	4940 (59.6%)
Low intensity without ezetimibe	534 (2.3%)	408 (2.1%)	139 (1.7%)
Statin + ezetimibe usage	1001 (4.3%)	1533 (7.7%)	759 (9.2%)
High intensity + ezetimibe	178 (0.8%)	405 (2.0%)	305 (3.7%)
Moderate intensity + ezetimibe	512 (2.2%)	696 (3.5%)	270 (3.3%)
Low intensity + ezetimibe	311 (1.3%)	432 (2.2%)	184 (2.2%)
Ezetimibe without statin usage	18 (0.1%)	16 (0.1%)	0 (0.0%)
Cardiovascular medications	*N* (%)	*N* (%)	*N* (%)
Renin‐angiotensin‐system‐acting agents	12 350 (52.6%)	15 122 (76.1%)	6604 (79.7%)
Diuretics	6296 (26.8%)	8430 (42.4%)	4391 (53.0%)
Aldosterone antagonists	640 (2.7%)	1236 (6.2%)	849 (10.2%)
Anticoagulants	1434 (6.1%)	2520 (12.7%)	1981 (23.9%)
Diabetes medications	*N* (%)	*N* (%)	*N* (%)
Glucose lowering drugs	6066 (25.9%)	10 238 (51.5%)	3248 (39.2%)
Insulins	827 (3.5%)	2003 (10.1%)	976 (11.8%)

Abbreviations: ASCVD, atherosclerotic cardiovascular disease; CV, cardiovascular; ESC, European Society of Cardiology; IQR: interquartile range; LDL‐C, Low‐density lipoprotein cholesterol; LLT, lipid‐lowering therapy.

### 3.2 LDL‐C control

**Figure 2:**
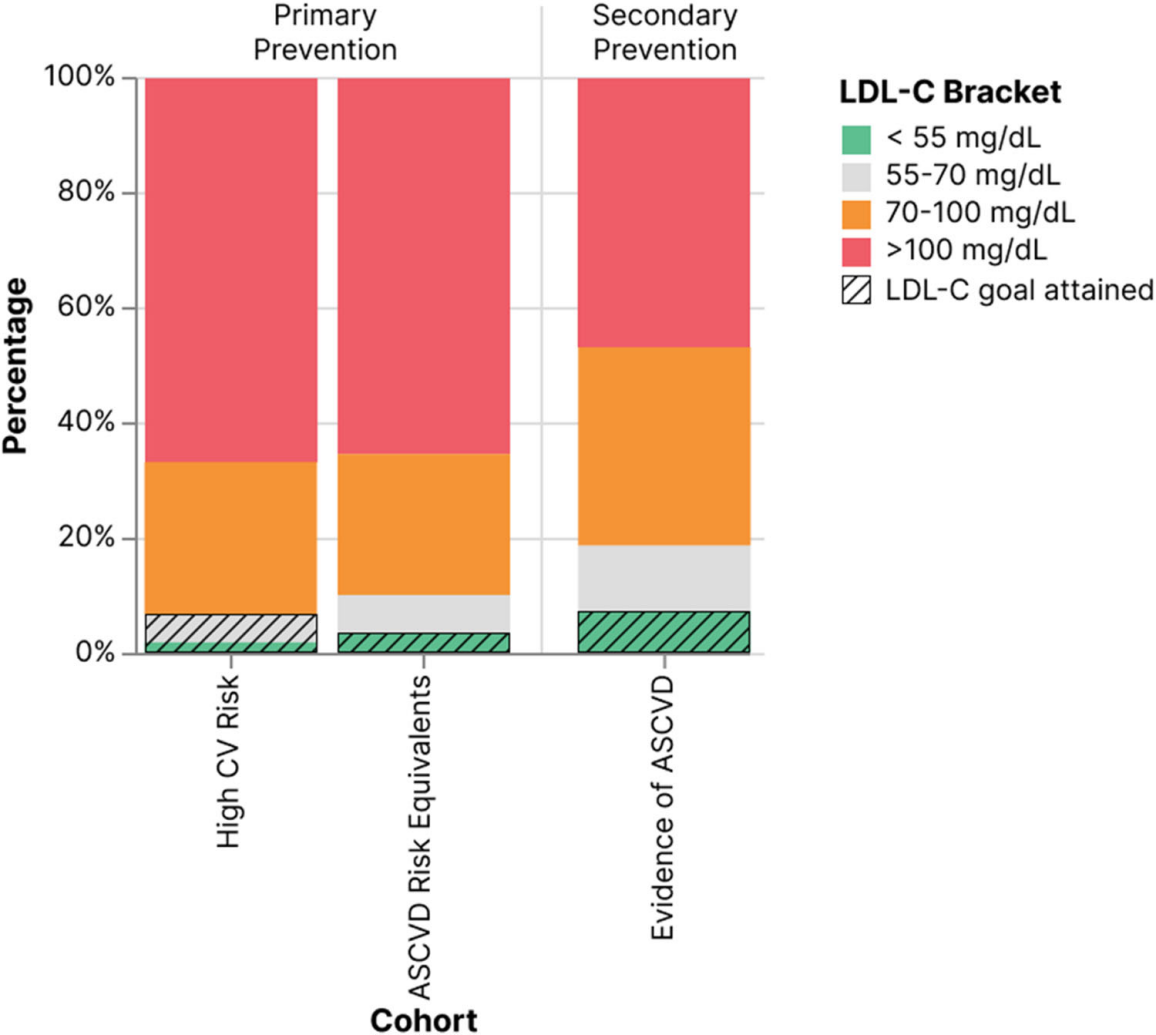
Low‐density lipoprotein cholesterol (LDL‐C) control according to the 2019 ESC/EAS guidelines for the high CV risk, ASCVD risk equivalents, and evidence of ASCVD cohorts.

We found that LDL‐C control, as per the ESC/EAS 2016 guidelines, was 32%, 10%, and 18% for the high CV risk, ASCVD equivalents, and evidence of ASCVD cohorts, respectively. Similarly, LDL‐C control considering the ESC/EAS 2019 guidelines, was 7%, 3%, and 7% for the same cohorts, respectively. Figure 2 presents the LDL‐C goal attainment in each cohort.

The discretized LDL‐C values of the high CV risk, ASCVD risk equivalence, and evidence of ASCVD cohorts are shown in Figures S1 and S2.

### 3.3 LLT use

**Figure 3:**
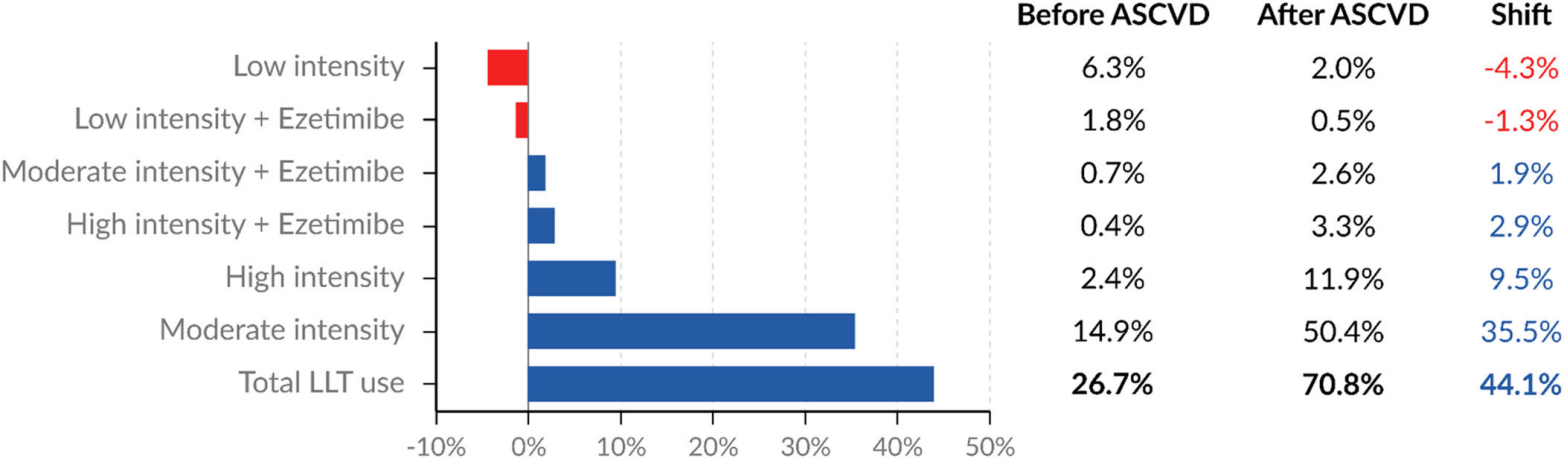
Lipid‐lowering therapy before and after ASCVD diagnosis.

The proportion of patients with no LLT prescribed differed among cohorts: 37% of those with high CV risk, 23% of those with ASCVD risk equivalents, and 15% of those with evidence of ASCVD had no LLT prescribed. Independent of the risk category, 73% of patients had no LLT prescribed before the event. After the ASCVD diagnosis, there was an increase of 36% in the use of moderate‐intensity statins in monotherapy, an increase of 10% in the use of high‐intensity statins in monotherapy, and the overall LLT prescription rate reached 85%. Figure 3 depicts the shift in the proportion of LLT (either statins or ezetimibe) prescribed before and after diagnosis of ASCVD.

### 3.4 Exclusive primary care versus cardiology follow‐up in ASCVD cohort

In the healthcare setting subanalysis of the 8288 patients with established ASCVD, 5262 were followed exclusively in primary care, and 3026 were also followed up in cardiology. Females constituted 55.3% in the primary care cohort compared to 35.9% in the cardiology cohort.

LDL‐C control varied between 15.3% in primary care versus 23.3% in cardiology based on ESC/EAS 2016 guidelines and 5.8% and 9.2%, respectively, for the 2019 guidelines. The median LDL‐C was higher in primary care at 102 mg/dL compared to 90 mg/dL in cardiology.

The cardiology cohort had an overall heightened prevalence of comorbidities, notably myocardial infarction at 62.7% versus 8.1% in primary care.

Regarding treatment patterns, the cardiology group had a lower number of patients (3.9%) without LLT, in contrast to 22.9% in primary care. High‐intensity statins, either alone or with ezetimibe, were more commonly prescribed in cardiology: 18.1% versus 7.7% for monotherapy and 6.1% versus 1.6% combined with ezetimibe.

For comprehensive details, refer to Table S6.

## 4 DISCUSSION

Considering the burden of ASCVD in Portugal, it is paramount to analyze the management of key risk factors, namely LDL‐C, to optimize CV prevention. This study revealed concerning findings about LDL‐C control and LLT prescription in high and very‐high CV risk populations. Poor LDL‐C control was seen across all cohorts when ESC/EAS 2016 guidelines were considered, with LDL‐C goal attainment rates of 32%, 10%, and 18% for high‐risk CV, ASCVD equivalent, and ASCVD cohorts, respectively. These rates were even lower when considering the ESC/EAS 2019 guidelines, which resulted in an additional 26%, 7%, and 11% of patients not achieving LDL‐C goal attainment, respectively.

In addition, our study revealed that 37% of high CV risk patients and 23% of ASCVD risk equivalent patients had no LLT prescribed. Nonetheless, 85% of patients with ASCVD had a prescription for any LLT. After ASCVD diagnosis, the use of moderate‐intensity statins in monotherapy increased by 36%, but the use of high‐intensity statins in monotherapy only increased by 10%.

There are two recent prospective registries^22^, ^23^ and a cross‐sectional multicentric analysis^13^ that give a similar insight on LLT prescription and LDL‐C control, although with better results than the ones we report. These differences could be explained by several important differences, such as the selected population, time of evaluation, and level of care at follow‐up.

The European Action on Secondary and Primary Prevention by Intervention to Reduce Events V of ESC‐EURObservational Research Programme (ESC‐EORP EUROASPIRE V survey) included 7824 patients with coronary artery disease (CAD) 6 months to 2 years after hospitalization of an acute coronary syndrome or revascularization, recruited from hospitals of 27 European countries in 2016–2017. Our study identified some notable differences in LDL‐C control and LLT prescription when compared to the EUROASPIRE V survey. In our study, a higher proportion of patients did not meet the LDL‐C goal (82% vs. 71%), and there was a lower prescription of high‐intensity statins (17% vs. 55%). However, our study included a broader spectrum of ASCVD, also including those with PAD and previous stroke, for which the adherence to guidelines in dyslipidemia management is even lower.^24^ Moreover, we considered all patients at any time point of follow‐up, many of them >2 years after the event and exclusively followed by primary care, which may have influenced adherence, LLT intensification, and less prescription of any LLT.^13^, ^25^ This is in line with the EUROASPIRE V survey, where 20.8% of the patients 6 months to 2 years after hospitalization had reduced the intensity of LLT prescribed at discharge or stopped it due to intolerance to LLT in 15.8% and by medical advice in 36.8%.

The 2020 EU‐Wide Cross‐Sectional Observational Study of Lipid‐Modifying Therapy Use in Secondary and Primary Care (DA VINCI study) included 3000 patients in primary prevention and 2888 in secondary prevention in 18 European countries. It found that only 25% of high CV risk patients, 11% of very‐high CV risk patients in primary prevention, and 18% of patients with established ASCVD achieved the LDL‐C values recommended by the 2019 ESC/EAS guidelines.^13^ As for LLT use, high‐intensity statin monotherapy was used in 20% and 38% of very high‐risk patients in primary and secondary prevention, respectively, compared to only 8.6% and 13.6% in our study. It is worth noting that the DA VINCI study was a registry study, and as such, the participating sites may have been biased in their selection of patients and in their approach to lipid‐modifying therapy.

The SANTORINI study included 9044 patients from March 2020 to February 2021, at high and very high CV risk, from 14 European countries. Only 24.0% of high and 18.6% of very‐high CV risk patients achieved LDL‐C risk‐based goals, and 23.5% and 21.1%, respectively, were not receiving any LLTs. In the SANTORINI study, physicians initially assigned CV risk at enrollment, while central assessments used the information from the study database. While this dual approach allowed for the physicians to reclassify patients when inconsistencies were found, underestimation of true risk might still persist. This finding reflects real‐world clinical practice, which often lags behind guideline recommendations, as highlighted by the study's authors. In our study, we utilized all available information in the EHR to calculate CV risk, which could potentially identify high and very high CV risk patients not evident to the physicians. This difference in risk assessment methodology may account for the slightly better results in LDL‐C attainment observed in the SANTORINI study compared to our study.^26^, ^27^

The earlier DYSIS and DYSIS II cohorts have revealed that a large portion of patients with very high CV risk fail to reach their LDL‐C goals, even when treated with LLT. In particular, only 21.7% of very high‐risk patients reached their LDL‐C goals in the DYSIS study, and in the DYSIS II study with patients with chronic CAD, only 29.6% of those on LLT and 8.3% of those untreated achieved LDL‐C levels <70 mg/dL.^28^, ^29^ In the Portuguese cohort of the DYSIS study, 62.9% of high‐risk patients and 57.3% of patients with ASCVD were not at the LDL‐C target levels recommended by the ESC. This cohort may, however, not reflect the current reality as the enrollment period occurred from April 2008 to February 2009, before the publication of the 2016 and 2019 updated ESC/EAS guidelines.^28^

In the USA, a 2011 study regarding 21 801 patients in the Veterans Affairs Health Care system in the Midwest region showed that LDL‐C goal attainment of <70 mg/dL in very high‐risk patients was just 41%.^30^ In addition, a treatment gap was identified in patients with elevated triglycerides, which was associated with even lower attainment of dual LDL‐C and non‐HDL‐C goals (13%).^30^ Similarly, Karalis et al. analyzed retrospective EHR of 23 408 California patients and found that in over 9950 CAD patients, only 37% of patients achieved LDL‐C control of <70 mg/dL.^31^ In the context of this study, it was observed that when patients were undergoing combination therapy, 41% of those receiving a combination of statin and ezetimibe and 46% of those on statin and niacin achieved the desired LDL‐cholesterol level of less than 70 mg/dL. However, by transitioning patients to a high‐potency statin, the attainment of the LDL‐cholesterol target of <70 mg/dL increased to 46%, and this figure rose substantially to 72% when combination therapy was employed.^31^

The more recent “Lipid Management in Portugal” (LATINO) study, a multicenter observational cohort study conducted in Portugal, characterized the CV risk profile and management of LDL‐C of patients. This study found that LDL‐C goals, according to 2019 ESC/EAS guidelines, were attained by 7% and 3% of patients, respectively, at high and very high CV risk. To achieve their targets, patients with uncontrolled LDL‐C levels who are at high and very high CV risk would need to decrease their LDL‐C levels by an average of 44% and 53%, respectively. Across all CV risk categories, moderate‐intensity statins were the most used, high‐intensity statins were prescribed in 5% of high CV risk patients and 10% of very high CV risk patients, and ezetimibe was used for 6% and 10% of these patients, respectively.^32^

The explanation for the poor results in LDL‐C control is multifactorial. There may be several reasons, such as patients' low adherence to treatment,^23^, ^33^, ^34^, ^35^ suboptimal or inadequate statin intensity,^24^, ^36^, ^37^, ^38^ concern with drugs adverse effects,^13^, ^36^ clinical inertia,^37^, ^38^ and possibly ineffective communication between physicians and patients.^36^ We believe that the low health literacy of the Portuguese population^39^ may also partly justify the poorer lipid control found when compared to the results of other European studies.^40^, ^41^

### 4.1 Strengths and limitations

A key strength of the PORTRAIT‐DYS study is the integration of EHR data from both primary and secondary healthcare units. As a result, our cohort is a close representation of the general CV risk population as observed in clinical practice. This study also benefits from having examined all EHR data from a period of approximately 20 years, offering robust and detailed insights into patient and family history.

A significant limitation of this study is its generalizability, as it presents data from a distinct population in the Northern region of Portugal. Nonetheless, the substantial number of patients included in the analysis from the overall population offers a detailed and unrestricted real‐world perspective of patient populations receiving primary and secondary care followed in similar contexts. In addition, we acknowledge that the utilization of LLT may be slightly overestimated since we could not confirm whether each prescription was actually dispensed or not.

### 4.2 Generalization of results

We characterized more than 95% of eligible patients at ULSM. Taking into account the high rate of ULSM utilization among the local residents, minimal migration rates of the population, and extension of the data collection period, we are confident these findings can be extrapolated to both the population served in this region and populations with similar characteristics and composition.

## 5 CONCLUSION

The findings from this study highlight a concerning gap between real‐world clinical practice and current guidelines for LDL‐C control and LLT prescription. A substantial proportion of high and very high‐risk patients have never received any LLT prescription, despite the fact that an LLT prescription, according to CV risk, is an important treatment opportunity.

Despite the availability of several options for effective LLT and the existence of generic high‐intensity statins, their broader use in higher‐risk populations and long‐term adherence are critical factors for the prevention of future CV events.

## AUTHOR CONTRIBUTIONS

*Conceptualization*: Cristina Gavina, Daniel Seabra‐Carvalho, Anastassia Anastassopoulou, Carla Teixeira, Jorge A. Ruivo, Élia Almeida, and Tiago Taveira‐Gomes. *Methodology*: Cristina Gavina and Tiago Taveira‐Gomes. *Software*: Tiago Taveira‐Gomes. *Validation*: Mariana Canelas‐Pais and Tiago Taveira‐Gomes. *Formal analysis*: Tiago Taveira‐Gomes. *Writing—original draft*: Ana Corte‐Real and Leonor Luz‐Duarte. *Writing—review and editing*: Cristina Gavina, Daniel Seabra‐Carvalho, Carlos Aguiar, Anastassia Anastassopoulou, Carla Teixeira, Jorge A. Ruivo, Élia Almeida, Ana Corte‐Real, Leonor Luz‐Duarte, Mariana Canelas‐Pais, and Tiago Taveira‐Gomes. *Supervision*: Cristina Gavina and Tiago Taveira‐Gomes.

## CONFLICT OF INTEREST STATEMENT

Cristina Gavina declares speaker and consulting fees from AstraZeneca, Bayer, BIAL, Boehringer‐Ingelheim, Daiichi Sankyo, Lilly, MSD, Novartis, and Novo Nordisk. Daniel Seabra‐Carvalho declares speaker fees from Daiichi Sankyo. Carlos Aguiar declares speaker and consulting fees from AstraZeneca, Bayer, BIAL, Daiichi Sankyo, Ferrer, MSD, Novartis, Novo Nordisk, and Servier. Anastassia Anastassopoulou, Carla Teixeira, Élia Almeida, and Jorge A. Ruivo are employees of Daiichi Sankyo. Tiago Taveira‐Gomes declares speaker and consulting fees from AstraZeneca, BIAL, Daiichi Sankyo, MSD, and Medinfar. Tiago Taveira‐Gomes holds shares in MTG. The results presented in this article have not been published previously in whole or part.

## Supporting information

Supporting information.

## Data Availability

All aggregate statistical results are incorporated into the article and its online supplementary material. Patient‐level data used in this study is not publicly available.
